# Prognosis and survival study in patients with gastric adenocarcinoma and its relationship with pRb expression alteration: A retrospective IHC‐based study

**DOI:** 10.1002/hsr2.1445

**Published:** 2023-07-26

**Authors:** Shokouh Taghipour Zahir, Seyyed Hossein Razavi, Farzan SafiDahaj, Koorosh Rahmani, Sajad Sadeghinejad‐Alamabadi

**Affiliations:** ^1^ Clinical and Surgical Pathology Shahid Sadoughi University of Medical Sciences Yazd Iran

**Keywords:** neoplasm grading, prognosis, Rb gene, stomach neoplasms, survival time

## Abstract

**Background and Objective:**

Among cancers, gastric cancer has the fifth highest incidence worldwide and is the third most common mortality factor, which may have been due to inadequate knowledge of its molecular pathogenesis. The retinoblastoma gene (RB1), a tumor suppressor gene, may have a role in gastric cancer. This research aims to assess Rb expression as a prognostic marker to obtain more insight regarding gastric cancer.

**Methods:**

This retrospective analytical study was done on 61 patients (45 males and 16 females) with gastric adenocarcinoma admitted from 2010 to 2012 in Shahid Sadoughi and Mortaz hospitals, Yazd, Iran. Demographic data, including age, gender, clinical signs and symptoms, and pathology reports, were retrieved from patients' hospital folders. Then, the altered Retinoblastoma gene expression was evaluated by immunohistochemistry studies. Acquired data were analyzed by SPSS software v.16. *p* < 0.05 was statistically considered meaningful.

**Results:**

In this study, the ratio of men to women was higher (2.81:1), and the mean age of patients was 62.44 years. About 90.2% of patients died during the study. There was no meaningful relationship between the presence of pRb, the intensity of staining, the percentage of staining with patients' age, gender, tumor grading, and survival rate (*p* > 0.05). There was only a meaningful relationship between the grade of tumors and survival rate (*p* = 0.039).

**Conclusion:**

Altered pRB expression is not common in gastric cancer and does not impact the survival and grading of tumors. Poorly differentiated tumors had an ominous outcome with the lowest survival time.

## 1 INTRODUCTION

Among cancers, gastric cancer has the fifth highest incidence worldwide and is the third most common mortality factor.^1^, ^2^, ^3^ In developing countries, over 70% of gastric cancers exist. Gastric cancer is more prevalent in men and has a survival of nearly 20% for 5 years, which may have been attributed to insufficient knowledge of its molecular pathogenesis; hence, there is no definitive chemotherapy regimen, and surgery is still a treatment option for choice.^1^, ^4^, ^5^, ^6^, ^7^, ^8^

Today, most cancer research is based on epigenetic and genetic modifications.^2^ The most frequent epigenetic change is the aberrant gene methylation that may induce tumor suppressor genes' deactivation (TSG). Learning about these changes is a priority to understand cancers more accurately.^2^, ^9^ Several forms of epigenetic changes or mutations have been found in gastric cancer, and various clinicopathological significances have been documented according to the methylation sites.^1^, ^2^, ^4^, ^9^, ^10^, ^11^

In many cancers, such as lung, bladder, breast, and brain, Rb (The retinoblastoma protein) and P53 suppression, which are essential TSGs, are seen. One of the cardinal cell cycle regulators in gastric cancer is the Rb gene pathway.^4^, ^9^, ^10^, ^12^, ^13^ Rb is a cell proliferation negative regulator that prohibits cells from advancing from the G1 phase into the S phase of the cell cycle. Another TSG called P16 is affected by Rb. P16 preserves Rb by blocking CKD4 and CKD6, contributing to the prevention and deactivation of Rb phosphorylation.^1^, ^2^, ^9^ Helicobacter pylori, which induces aberrant methylation of the P53‐Rb pathway by several mechanisms, especially oxygen radicals, is an acquired risk factor for gastric cancer.^2^, ^9^, ^12^, ^14^, ^15^ Compared to normal or pre‐malignant tissues, some studies have documented decreased Rb gene product (pRb) in gastric cancer; therefore, it has diagnostic value in the early stages of the disease.^11^, ^13^, ^16^ At the same time, studies in pre‐malignant states report increased Rb expression.^14^ There are also reports of gastric cancer that suggest Rb gene deletion.^1^, ^10^

Some data indicate that Rb expression induces a better prognosis in gastric cancer, while some findings suggest that it has no prognostic significance^13^, ^17^; thus, to achieve better treatment for gastric cancer, further research is required to clarify the interaction between the Rb gene and pRb and patient prognosis.

## 2 METHODS

This research was a retrospective and cross‐sectional descriptive‐analytical study of all patients with gastric adenocarcinoma admitted during 2010−2012 to Shahid Sadoughi and Mortaz hospitals in Yazd, Iran. Patients' missing details (e.g., survival, cause of death, and inaccurate telephone number) and deaths due to causes other than gastric cancer were omitted from the criterion.

Demographic information from the hospital ward archive, including age, gender, paraclinical information, surgery report, and pathology report, was retrieved. Then, appropriate gastric tissue blocks were chosen by evaluating hematoxylin and eosin stained slides in the pathology ward archive. Selected gastric tissue blocks preserved in paraffin were cut again, stained by immunohistochemistry (IHC), and then evaluated for the Rb gene.

### 2.1 Immunohistochemistry evaluation

Cancerous tissue samples were picked and sliced into 4‐μm‐thick pieces from paraffin‐embedded blocks. In xylene, they were de‐paraffinized, dehydrated by graded alcohols, and placed in 0.1% hydrogen peroxide to quench endogenous peroxidase activity. In a 10 mmol sodium citrate buffer (10 mmol/L, pH 6.0), antigen retrieval was carried out using a 750 W microwave oven for 15 min. To avoid the nonspecific binding of antibodies, the sections were blocked with 10% normal goat serum for 30 min at room temperature. Inside a humidified chamber at 4°C overnight, the slides were incubated with anti‐human Rb monoclonal antibody (Biogen; DBS; Mouse Anti Retinoblastoma Antibody; 31703458570). The sections were then incubated at 37°C for 30 min with a biotin‐labeled goat secondary anti‐rabbit antibody (Master polymer plus HRP), followed by the streptavidin‐biotin horseradish peroxidase complex reaction. The reaction products were detected by immersing the slides in a freshly formulated diaminobenzidine solution for 10 min before dehydration and mounting and by counterstaining them with hematoxylin.

**Figure 1:**
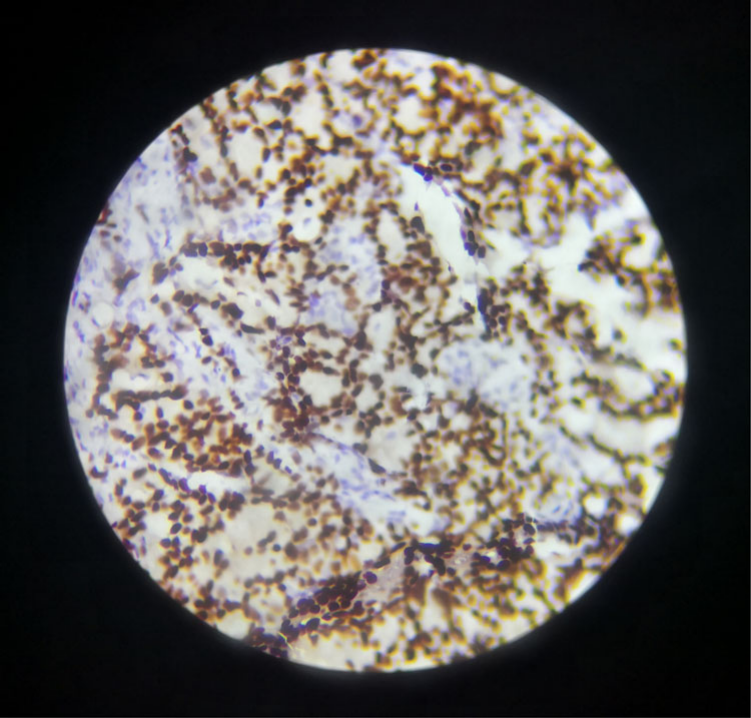
Strongly positive staining of neoplastic cells for Rb (×20, IHC staining).

**Figure 2:**
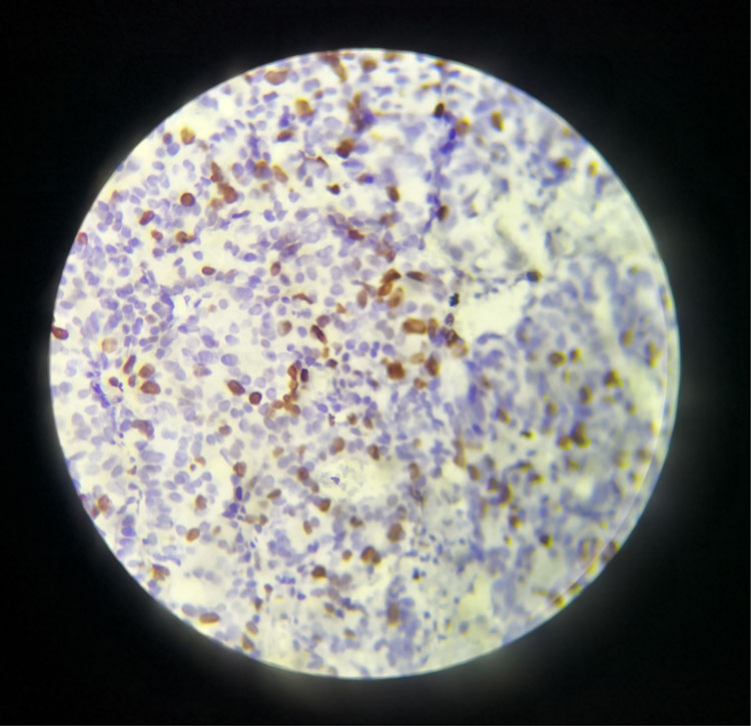
Moderately positive staining of neoplastic cells for Rb (×20, IHC staining).

All slides were assessed under low magnification (×4) and then confirmed under high magnification (×20 and ×40) by light microscopy scanning the whole tissue specimen. A professional pathologist, blinded to the study design, assessed the staining severity. The immunostaining for RB protein was considered positive if there was brown nuclear staining of the neoplastic cells. So, normal and aberrant pRB expressions were determined for invasive carcinoma cases. Normal pRB expression was reflected by nuclear staining in all areas of tumor tissue with cell‐to‐cell heterogeneity and variable staining intensity. pRB expression was considered aberrant if the whole section or major focal areas within the section showed no nuclear staining with preserved reactivity in adjacent non‐neoplastic tissue. Samples were graded in three ways: positive/negative for the mutated Rb gene and stained tissue intensity (negative, weak, and strong) (Figures 1 and 2). The intensity of the staining of malignant cells (negative, weak, and strong) was quantitatively interpreted. The interpretation of intensity was based on positive and negative controls. Therefore, if the color intensity was like positive control, it was considered a strong intensity. If it did not have a color, it was considered negative. It was considered weak if the staining intensity was slightly higher than the negative control. The percentage of stained cells was calculated by counting a hundred malignant cells and the number of stained cells. (negative(0), < 5% mild, 5−15% moderate, and above 15% as a high percentage).

### 2.2 Statistical analysis

All data was put into the program SPSS v.16 (SPSS, Inc.) and analyzed by *T*‐test, *χ* ^2^ test, and ANOVA test. *P* Values lower than 0.05 were considered significant.

## 3 RESULTS

Of the 61 census‐studied patients, 45 (73.7%) were male, and 16 (26.3%) were female (male to female ratio was 2.81). The youngest patient was 21 years old, and the oldest was 91. For males, the average age was 64.13 year old, 57.68 year old for women, and 62.44 year old for all patients. Most patients (37 individuals) were 50 to 80 years of age. Fifty‐five patients (90.2%) had died within 5 years of diagnosis due to gastric cancer, and six patients (9.8%) were still alive.

As shown in Table 1, no significant relationship was shown between the altered Rb expression and gender (*p* = 0.272). There was also no relationship between age and the pRb expression (*p* = 0.57). Following block staining, 43 patients (70.5%) did not show positive stains, and 18 patients (29.5%) had positivity for pRb IHC‐staining. Eight (17.8%) of male patients had poorly stained tissue severity, and seven (15.6%) had strong intensity. No relationship between gender and the staining intensity of the tissue was found based on these results (*p* = 0.513). The average age of patients who do not have a mutant pRb is 61.67. As shown in Table 2, 11 tumors were well differentiated (18%), 21 were moderately differentiated (32.8%), and 30 were poorly differentiated (49.2%). There was no significant gender‐tumor grading association (*p* = 0.988). In poorly differentiated tumors, 18 (60%) tumors had no altered pRb expression, and 12 (40%) tumors had a pRb mutation. This data found no relationship between grading and the pRb mutation (*p* = 0.064). Thirty‐nine patients (90.7%) with pRb mutation died from gastric cancer. There was no relationship between the pRb mutation and the 5‐year survival of patients (*p* = 0.829). Two patients with well‐differentiated tumors (18.2%), four patients with moderately differentiated tumors (20%), and no one with poorly differentiated tumors was alive after 5 years (Details are shown in Table 1). However, the relationship between 5‐year survival and tumor grading was meaningful (*p* = 0.039), that the less differentiation, the worse survival was seen.

**Table 1:** Clinicopathological features of the patients with gastric adenocarcinoma in terms of Rb mutation.

Variables	Mutated Rb		Rb intensity			Rb %				Total
	Neg	Pos	Neg	Low	High	0	<5%	5−15%	>15%	
Gender										
Male	30 (66.7%)	15 (33.3%)	30 (66.7%)	8 (17.8%)	7 (15.6%)	30 (66.7%)	5 (11.1%)	5 (11.1%)	5 (11.1%)	45 (100%)
Female	13 (31.3%)	3 (18.8%)	13 (81.3%)	2 (12.5%)	1 (6.3%)	13 (81.3%)	2 (12.5%)	1 (6.3%)	0	16 (100%)
*p* Value (test)	0.272 (*χ* ^2^)		0.513 (*χ* ^2^)			0.487 (*χ* ^2^)				‐
Mean Age	61.6	64.2	61.67	68.4	59.12	61.67	67.28	65	59.2	62.44
*p* Value (test)	0.57 (*T* test)		0.425 (ANOVA)			0.798 (ANOVA)				‐
Survival										
Dead	39 (90.7%)	16 (88.9%)	39 (90.7%)	8 (80.1%)	8 (100%)	39 (90.7%)	6 (85.7%)	5 (83.3%)	5 (100%9)	55 (90.2%)
Alive	4 (9.3%)	2 (11.1%)	4 (9.3%)	2 (20%)	0	4 (9.3%)	1 (14.3%)	1 (16.7%)	0	6 (9.8%)
*p* Value (test)	0.829 (*χ* ^2^)		0.359 (*χ* ^2^)			0.794 (*χ* ^2^)				‐
Grade										
Well	7 (63.6%)	4 (36.4%)	7 (63.6%)	3 (27.3%)	1 (9.1%)	7 (63.6%)	3 (27.3%)	0	1 (9.1%)	11 (100%)
Moderate	18 (90%)	2 (10%)	18 (90%)	2 (10%)	0	18 (90%)	1 (5%)	1 (5%)	0	20 (100%)
Poor	18 (60%)	12 (40%)	18 (60%)	5 (16.7%)	7 (23.3%)	18 (60%)	3 (10%)	5 (16.7%)	4 (13.3%)	30 (100%)
*p* Value (test)	0.064 (*χ* ^2^)		0.089 (*χ* ^2^)			0.109 (*χ* ^2^)				‐
Total	43	18	43	10	8	43	7	6	5	61

**Table 2:** Distribution of the patients' demographic data according to the tumor grading and survival.

Variables	Grade			Survival		Total
	Well	Moderate	Poor	Dead	Alive	
Gender						
Male	8 (17.8%)	15 (33.3%)	22 (48.9%)	40 (88.9%)	5 (11.1%)	45 (100%)
Female	3 (18.8%)	5 (31.3%)	8 (50%)	15 (93.8%)	1 (6.3%)	16 (100%)
*p* Value (test)	0.988 (*χ* ^2^)			0.575 (*χ* ^2^)		‐
Mean Age	66.18	62.35	61.13	62.7	60	62.44
*p* Value (test)	0.689 (ANOVA)			0.70 (*T* test)		‐
Total	11	20	30	55	6	61

Due to gastric cancer, all patients with high‐intensity‐stained tissue had also expired. Therefore, there was no relationship between the severity of staining and 5‐year survival (*p* = 0.359). Forty out of 45 male patients (89.9%) and 15 out of 16 female patients (93.7%) died from cancer because there was no gender‐to‐survival relationship (*p* = 0.575). Also, there was no correlation between patient age and survival (*p* = 0.700) (Details are shown in Table 2).

## 4 DISCUSSION

Gastric cancer, especially among older males, remains among the most frequent and fatal cancers worldwide. We evaluated the altered Rb gene expression in the present study and its connection to gender, age, tumor grade, and survival in gastric cancer.

Mousavi et al. and other studies reported that with a ratio of 1.2−6.3, men grow gastric cancer rather than women.^5^, ^6^, ^7^ In our study, males had more gastric cancer than females (2.81:1). Most Crew et al. study patients were between 50−70 year old and 60−80 year old in the Krejes et al. research.^18^, ^19^ In our survey, most patients were aged 50−80, and the average age was 62.4 year old. There was no relationship between pRb mutation, age, and gender in the current study, as in Chou et al. and Song et al.^3^, ^20^

There are distinct findings from research performed on the Rb gene. Some experiments indicate increased expression of Rb, and some display reduced expression. The pRb was reduced by 46% in Yunemura et al. 48.9% in Ogawa et al. 56.3% in Song et al. 40% in Kishimoto et al., and 33% in Feakins et al. studies.^3^, ^17^, ^21^, ^22^, ^23^

Reduced pRb compared to normal tissue was also reported by Miao et al.^16^ More than 60% altered pRb has been reported by Abdel‐Aziz et al. and Chou et al. and concluded that Rb mutation is a prevalent occurrence in gastric cancer. However, Constancia et al. suggest that Rb alteration is not widely seen in gastric cancer.^20^, ^24^, ^25^

Analysis by Chen et al. showed decreased mRNA of the Rb gene and concluded that decreased Rb expression is associated with gastric cancer.^26^ In comparison to the above findings, some studies have shown high intact pRb (up to 87%) or increased Rb expression in gastric cancer and say that this increased expression in early gastric cancer could have diagnostic values.^13^, ^14^, ^16^, ^27^ In the current study, only 29.5% of cells had altered Rb gene expression, indicating that Rb mutations are less frequent in gastric cancer than in the Constancia et al. study.^25^

Further investigations are required to determine the Rb gene correlation to the grade and stage of tumors.^24^ Chou et al. conclude that there is a significant relationship between altered pRb and tumor stage,^20^ and Song et al. say fewer vascular invasions in intact pRb cells. The research of Chen et al. reveals that Rb expression reduces cell invasion.^3^, ^28^ The only prognostic factor for lymph node invasion was Rb expression, as stated by Sungon et al.^29^ There was no relationship between Rb expression or altered, stained pRb intensity and tumor grading in some studies.^13^, ^17^, ^20^, ^21^, ^24^ However, Mattioli et al. stated that some Rb pathway proteins (pRb2/p109) were associated with low‐grade tumors.^30^ We found no relationship between mutated Rb gene expression and tumor grading, as Yonemura et al. and other similar studies (*p* = 0.64).

Data regarding the Rb gene and its prognosis relationship varies, some indicating that Rb has a prognostic value, while some deny this.^13^ Kishimoto et al. indicated that the prognosis is correlated with Rb.^22^ Studies by Yonemura et al., Ogawa et al. and Kouraklis et al. imply that tumors that contain pRb have a higher prognosis and that Rb expression has a prognostic value.^17^, ^21^, ^31^

Feakins et al. and Song et al. also claim a poorer prognosis for tumors that produce less pRb.^3^, ^23^ In their study, Chou et al. demonstrated that tumors in which less stained cells (<50%) were seen for altered pRb had a worse 5‐year survival than those with more stained cells (>50%). There is a negative association between Rb expression and survival in univariate analysis, although there is no relationship in multivariate analysis. They argued that the Rb gene is not an independent prognostic factor.^20^ This research shows that the pRb expression alteration may not be a prognostic factor (*p* = 0.829) and would not contribute to 5‐year survival. Tumor grading is the only prognostic factor in the current analysis; thus, survival drops with lower differentiations (*p* = 0.039).

The limitation of our study was the small sample size and the loss of some samples due to lack of access to patients or lack of some considered variables in patients' files.

## 5 CONCLUSION

The presented data indicates that gastric cancer is more prevalent in males than females, but age, gender, altered pRb expression, and survival are not correlated. In 29.5% of tumors, the altered pRb expression is shown and is thus not a prevalent phenomenon in gastric cancer. Furthermore, there is no association between altered pRb expression and patient prognosis. The degree of altered pRb expression and the proportion of mutated cells do not significantly impact prognosis. However, tumor grading is linked to patient survival, with lower differentiation resulting in reduced 5‐year survival rates. Altered pRb expression, staining intensity, and the percentage of mutated cells do not correlate with tumor grading. More extensive sampling is necessary to identify the prognostic value of altered pRb in gastric adenocarcinoma neoplastic cells and other factors that may contribute to disease progression. Additionally, a genomic‐level examination is advised. Further research on gene mutation's influence on angiogenesis and vascular invasion and its potential impact on treatment sensitivity or resistance would be beneficial.

## AUTHOR CONTRIBUTIONS

**Shokouh Taghipour Zahir**: Conceptualization; formal analysis; supervision; validation; writing—original draft; writing—review and editing. **Seyyed Hossein Razavi**: Conceptualization; investigation; project administration; writing—original draft; writing—review and editing. **Farzan SafiDahaj**: Formal analysis; methodology; project administration; writing—original draft; writing—review and editing. **Koorosh Rahmani**: Investigation; project administration; writing—original draft; writing—review and editing. **Sajad Sadeghinejad‐Alamabadi**: Investigation; methodology; project administration; writing—original draft; writing—review and editing.

## CONFLICT OF INTEREST STATEMENT

The authors declare no conflict of interest.

## ETHICS STATEMENT

This research was analyzed and obtained ethnic code by the Yazd Shahid Sadoughi Medical Science University ethnic commission (IR.SSU.MEDICINE.REC.1396.37). The authorization to view patient records and gather the required data was collected from medical centers. A trained medical student telephoned the family of patients to obtain survival information. During and after the research, the patient's details were confidential.

## TRANSPARENCY STATEMENT

The lead author Sajad Sadeghinejad‐Alamabadi affirms that this manuscript is an honest, accurate, and transparent account of the study being reported; that no important aspects of the study have been omitted; and that any discrepancies from the study as planned (and, if relevant, registered) have been explained.

## Data Availability

Data available on request from the authors.
